# A novel hybrid scale for the assessment of cognitive and executive function: The Free‐Cog

**DOI:** 10.1002/gps.5454

**Published:** 2020-11-16

**Authors:** Alistair Burns, Judith R. Harrison, Catherine Symonds, Julie Morris

**Affiliations:** ^1^ University of Manchester Manchester UK; ^2^ Cardiff University Cardiff UK; ^3^ Greater Manchester Mental Health NHS Foundation Trust Manchester UK

**Keywords:** cognitive scale, cognitive test, dementia, diagnosis, Free‐Cog

## Abstract

**Background:**

Scales measuring cognitive and executive functions are integral to the assessment and management of patients with suspected cognitive impairment. Some of the most commonly used cognitive tests are now subject to copyright restrictions. Furthermore, no existing scale assesses both executive and cognitive abilities.

**Aims:**

We aimed to develop and validate a novel hybrid scale for use in clinical practice which integrate measures of cognition and executive abilities (‘Free‐Cog’).

**Methods:**

The instrument was devised through a national collaboration including health professionals, those with lived experience of dementia and researchers. Following ethics committee approval, the Free‐Cog was assessed in 25 real‐world clinical settings across England, Wales and Scotland. It was compared to three other cognitive tests routinely administered in clinical practice: the Mini‐Mental State Examination (MMSE), the Montreal Cognitive Assessment (MOCA), and the Addenbrooke's Cognitive Examination (ACE).

**Results:**

The Free‐Cog was tested in 960 patients with clinical diagnoses of dementia, Mild Cognitive Impairment (MCI), and normal controls. Similar to the MMSE, MOCA and ACE, it discriminated well between the three groups (*p* < 0.001). It correlated well with the other instruments. Using a receiver operating characteristic curve analysis, Free‐Cog achieved an Area Under Curve of 0.94 for dementia versus controls, 0.80 for MCI versus controls and 0.77 for dementia versus MCI. A version of the tool adapted for telephone consultation, the Tele Free‐Cog, also discriminated well between patient groups.

**Conclusions:**

Free‐Cog is a non‐proprietary, empirically derived, concise assessment. Uniquely, it combines cognitive and executive function questions in the one instrument. It could be used to inform the assessment of people presenting with cognitive impairment and is available to anyone interested in trialling it.

## 1 INTRODUCTION

### 1.1 Cognitive and functional scales

Cognitive and functional scales are an essential part of the assessment and management of people with suspected cognitive impairment. Although there are numerous tests in the literature, the most well‐known is the Mini‐Mental State Examination (MMSE).^1^ Published almost 50 years ago, it was designed to operationalise cognitive assessment in the busy acute hospital setting.^2^ Reliable, quick and straightforward to administer, the MMSE became a key tool both for frontline clinicians and researchers. Experienced clinicians even predict the MMSE score from a general conversation.^3^ However, the MMSE is not sensitive to early cognitive changes.^4^ Other tools, such as the Montreal Cognitive Assessment (MOCA),^5^ were subsequently developed to detect more subtle impairment. Like the MMSE, the MOCA is a concise test that is commonly used in clinical practice. Access to both the MOCA and MMSE are now subject to copyright restrictions, which has caused concern among many clinicians.^6^, ^7^

**Key points**

- Assessment of cognitive and executive function are both important in the assessment of patients with suspected dementia
- Free‐Cog combines for the first time in one scale these two elements
- Free‐Cog has acceptable metrics when compared to other similar assessments

None of the current cognitive tests include assessment of executive abilities. The effects of executive dysfunction (in terms of functional abilities) are usually assessed in a parallel process, exploring basic living activities and instrumental activities as markers of early and late stage dementia, respectively.^6^, ^7^, ^8^, ^9^, ^10^ Given the increasing prevalence of dementias and the urgent need for breakthroughs in dementia research, the need for accessible assessment instruments has never been greater. We had two objectives. We aimed to develop a scale which (i) combined both functional and executive assessments and (ii) would be free‐to‐use.

### 1.2 Development of Free‐Cog

The Free‐Cog development project was a national collaboration including academics at the Universities of Manchester and Cardiff, the Royal College of Psychiatrists, people with lived experience of dementia and their carers, and frontline clinicians with experience of using assessment scales. Initial versions were based on the expert opinion of experienced clinicians from focus groups hosted by the Royal College of Psychiatrists. We sought feedback from those with lived experience, their carers, academics working in dementia and frontline clinicians, resulting in iterative modification over 3 years. Early changes improved the discrimination of the cognitive items and the acceptability of the functional questions. The current study used the final revised version of the Free‐Cog.

### 1.3 Description of Free‐Cog

The Free‐Cog is reproduced in the Appendix S1. The first section assesses cognitive function and comprises 12 domains. It begins with a conversational domain which addresses general knowledge, designed to make patients feel comfortable and help establish rapport. Subsequent domains assess orientation, memory, calculation, attention, visuospatial function, language and fluency. Only the fluency task is timed. The maximum score for this section is 25. The second section assesses executive function. It includes five domains covering activities of daily life, including social functioning, travel, self‐care and safety at home. The maximum score for this section is 5. The total maximum score is 30. Unlike other similar instruments, we do not provide detailed instructions for administration in a separate document. Rather, essential scoring guidance is included with the test domains on one page of A4 paper. This was thought to be more suitable for busy clinical environments.

The present study aims to validate the Free‐Cog against gold‐standard brief cognitive assessments.

## 2 SUBJECTS AND METHODS

The authors assert that all procedures contributing to this work comply with the ethical standards of the relevant national and institutional committees on human experimentation and with the Helsinki Declaration of 1975, as revised in 2008. All procedures involving human subjects/patients were approved by the local NHS Research Ethics Committee (IRAS project id: 227062). Through our collaborative network we identified 25 sites across the United Kingdom (see Appendix S1). We recruited from 17th January 2018 to the 31st August 2019. We aimed to obtain a sample that was representative of the UK population in outpatient secondary care services for older adults with cognitive impairment. We included any participants who were referred for a memory assessment or had a diagnosis of dementia/memory impairment as dementia/mild cognitive impairment (MCI) cases. Any participants with no diagnosis of dementia/memory impairment and no self‐reported concerns of memory impairment were included as healthy controls. We excluded participants under 18 years old.

Most participants were recruited in frontline healthcare settings such as non‐specialist memory clinics, community mental health teams for older people and other services dealing with people with suspected dementia. A minority of participants were assessed in a research environment and were also taking part in dementia genetics studies.

Written informed consent was obtained from all participants. Issues of confidentiality, feedback to patients, particularly controls who may have scored low on the tests, and issues of mental capacity were dealt with according to the ethics committee judgement.

We recorded clinical diagnoses which were confined to three basic categories: dementia (Alzheimer's disease, frontotemporal dementia, Lewy body dementia, vascular dementia, Parkinson's disease dementia, early onset dementia, other dementias, mixed Alzheimer/vascular dementia); MCI; or controls (defined as people without any symptoms suggestive of cognitive decline). The comparator standard tests were: the MMSE,^1^ the MOCA,^5^ and the Addenbrooke's Cognitive Examination (ACE).^11^

There was no specific training or written guidance provided for the Free‐Cog. The centres were considered expert in the administration and scoring of cognitive tests. The protocol asked clinicians to use their clinical judgement when interpreting answers to questions and encouraged to follow their usual clinical procedures. For example, the participant would have a normal assessment in the clinic, including the cognitive tests the clinic usually applied. The Free‐Cog was then administered at the same time or within one week of the standard assessment.

Data were collected anonymously. Only age, gender and diagnosis were available to the research team. Analyses of covariance with Bonferroni corrections were used to compare the cognitive measures between the three subject groups, adjusting for age and gender. There was minimal difference between the age and gender‐adjusted means and the observed means for all measures and therefore the latter are presented as descriptive statistics in the Tables. Spearman correlation coefficients were used to measure the similarity in ranking of individuals according to the different cognitive scores, and the predictive power of the measures were compared using receiver operating characteristic (ROC) curve analysis. The discriminative ability of the individual items of Free‐Cog was assessed using multivariable logistic regression analysis.

## 3 RESULTS

In total, 960 participants took part in the study. Participant characteristics are shown in Table 1. Twelve subjects were excluded from the analysis: ten because a diagnosis was omitted; two remained under investigation.

**TABLE 1:** Participant characteristics

Characteristic	Age (SD)	Female N (%)
Dementia (*N* = 465)	77.3 (8.7)	203 (44)
Mild cognitive impairment (*N* = 128)	76.0 (9.7)	63 (49)
Control (*N* = 355)	63.1 (14.4)	250 (70)

Table 2 shows the total Free‐Cog score (out of 30), the Free‐Cog cognitive score (out of 25), the Free‐Cog executive score (out of 5), and the other scales for each participant group. For each individual, scores were obtained on at most two alternative scales, hence the group sizes for these scales are smaller than those for Free‐Cog. The dementia group included a minority of high scores, however the diagnoses were assigned by the teams and no other data was available to the research team (e.g., to indicate individuals with genetic diagnoses of familial Alzheimer's disease who were pre‐symptomatic).

**TABLE 2:** Test scores on the Free‐Cog, MOCA, MMSE and ACE

Tests	Control (SD, range)	MCI (SD, range)	Dementia (SD, range)
Total Free‐Cog (*N* = 948)	28.1^a^ ^,^ ^b^ (1.9, 17–30)	25.1^c^ (3.2, 15–30)	20.0 (5.9, 0–30)
Cognitive Free‐Cog (*N* = 493)	23.1^a^ ^,^ ^b^ (1.6, 18–25)	20.5^c^ (3.2, 12–25)	15.8 (5.4, 0–25)
Executive Free‐Cog (*N* = 493)	4.8^b^ (0.4, 3–5)	4.7^c^ (0.6, 2–5)	4.1 (1.3, 0–5)
MOCA (*N* = 525)	27.8^a^ ^,^ ^b^ (2.1, 16–30)	23.4^c^ (3.6, 14–30)	17.1 (6.3, 0–30)
MMSE (*N* = 158)	28.7^b^ (1.8, 20–30)	26.2^c^ (3.3, 19–30)	21.6 (6.5, 1–30)
ACE (*N* = 306)	84.2^b^ (23.9, 25–100)	73.9^c^ (22.4, 13–98)	63.5 (21.2, 6–99)

Abbreviations: ACE, Addenbrookes' Cognitive Assessment; MOCA, Montreal Cognitive Assessment; MMSE, Mini‐Mental State Examination; SD, standard deviation.

^a^ Control versus MCI; *p* < 0.05.

^b^ Control versus Dementia; *p* < 0.05.

^c^ MCI versus Dementia; *p* < 0.05.

For the total Free‐Cog score and its two component scores there was an overall significant difference between the three groups (*p* < 0.001), and specifically between the Control and Dementia group (*p* < 0.001), and the MCI and Dementia group (*p <* 0.001). For the total score and the cognitive component score there was also a significant difference between the control and MCI group (*p* < 0.005).

There were also significant overall differences between the three groups for the MOCA, MMSE and ACE scores (*p* < 0.001), and specifically between the Control and Dementia group (*p* < 0.001), and the MCI and Dementia group (*p* < 0.001). For the MOCA scale there was also a significant difference between the control and MCI group (*p* < 0.001).

Free‐Cog total also showed similar differences between groups for the matching respective subgroups of individuals with MOCA, MMSE and ACE data as for the whole cohort (data not shown). Therefore, the Free‐Cog, MOCA and MMSE appear to be similar in their discrimination between the three groups.

For patients with MCI or dementia, the correlation between Free‐Cog and MOCA (*n* = 301) was 0.85 (*p* < 0.01); with the MMSE (*n* = 98) was 0.84 (*p* < 0.001) and with the ACE (*n* = 237) was 0.62 (*p* < 0.01). This shows a high degree of association between the rankings of individuals according to the Free‐Cog total and the rankings according to the three cognitive measures. This high degree of association was present for the separate groups of patients with MCI and for patients with dementia.

**FIGURE 1:**
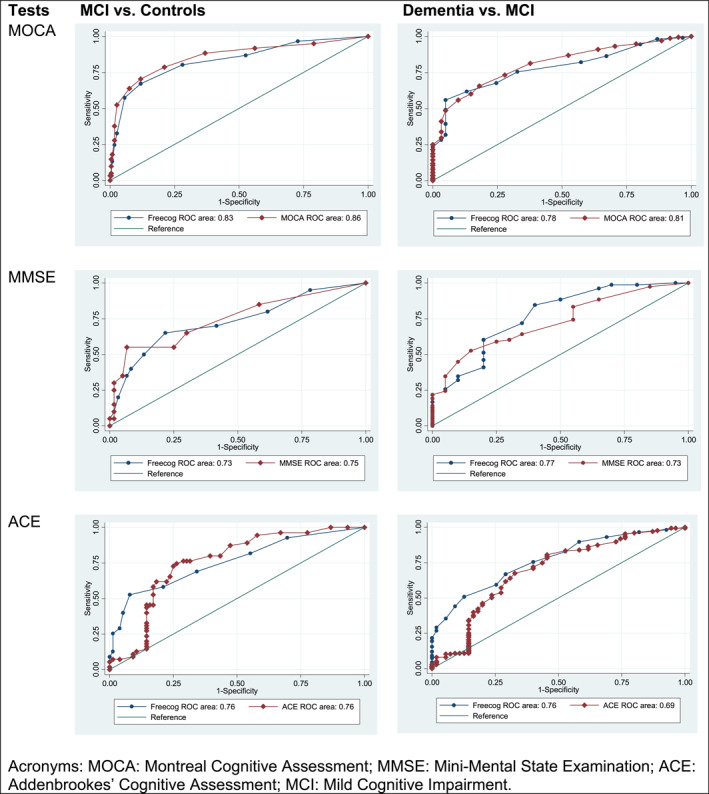
ROC curve analysis, showing the AUC for dementia versus MCI and MCI versus controls for the Free‐Cog compared to the MOCA, MMSE and ACE. ACE, Addenbrookes' Cognitive Assessment; AUC, area under curve; MCI, mild cognitive impairment; MMSE, Mini‐Mental State Examination; MOCA, Montreal Cognitive Assessment; ROC, receiver operating characteristic

Using ROC curve analysis, the Free‐Cog total score showed the same discriminatory ability as the other cognitive assessments (MOCA, ACE and MMSE) to distinguish between people with MCI and dementia and between controls and people with MCI (Figure 1). The Free‐Cog produced satisfactory sensitivity and specificity rates (Table 3).

**TABLE 3:** Sensitivity and specificity for Free‐Cog total score (*N* = 948)

	Sample cut‐points to identify dementia/MCI	Sensitivity %	Specificity %	AUC
Dementia versus control	≤26	83	80	0.94
	≤27	89	72	
	≤28	94	58	
MCI versus control	≤27	75	70	0.80
	≤28	84	46	
	≤29	94	27	
Dementia versus MCI	≤24	77	63	0.77
	≤25	83	45	
	≤26	89	36	

Abbreviations: AUC, area under curve; MCI, mild cognitive impairment.

The group was divided into those with Alzheimer's disease (*N* = 340) and those with other dementias (*N* = 125). The Alzheimer group scored lower than the other dementia group on the total Free‐Cog (mean score 19.2, standard deviation [SD] 6, range 0–30 compared to a mean score of 22.3 SD 5.2, range 2–30). These two groups were compared to those with controls and MCI. There were significant differences (*p* < 0.05) between all four groups on the total and cognitive Free‐Cog total scores. On the executive scores, the two dementia groups were different to the controls and MCI groups but that no differences between the two dementia groups. The results for the other three cognitive tests were similar (data not shown).

Using the matching subgroups of individuals with results from the MOCA (*n* = 525), ACE (*n* = 306) and MMSE (*n* = 158), the Free‐Cog had better discrimination between Alzheimer's and non‐Alzheimer's dementia (Free‐Cog *p* < 0.001 and MOCA *p* = 0.013; Free‐Cog *p* = 0.001 and ACE *p* = 0.59, Free‐Cog *p* < 0.001 and MMSE *p* = 0.003).

Data on individual components of Free‐Cog were obtained for 107 subjects (64 with dementia, 20 with MCI and 23 controls). Logistic regression analysis identified the component ‘memory‐recall’ as the sole significant independent discriminator between the MCI and control groups, the components ‘calculation’ and ‘visuospatial’ as significant independent discriminators between the MCI and dementia groups, and the components, ‘memory recall’, ‘calculation’, ‘visuospatial’ and ‘general knowledge’ as significant independent discriminators between the dementia and control groups.

We tested a revised scale score to facilitate telephone consultations, the Tele Free‐Cog This excludes three components, ‘visuospatial’ (clock face) ‘language’ (name ear/fingernail) and ‘write a sentence’, has a total score of 24. Among those with subcomponent scores available (*N* = 107), the Tele Free‐Cog, showed reasonable sensitivity and specificity, comparable to the Free‐Cog (Table 4).

**TABLE 4:** Sensitivity and specificity for Tele Free‐Cog score (*N* = 107)

	Sample cut‐points to identify dementia/MCI	Sensitivity %	Specificity %	AUC
Dementia versus control	≤19	87	100	0.95
	≤20	90	83	
	≤21	94	65	
MCI versus control	≤20	70	83	0.85
	≤21	85	65	
	≤22	90	26	
Dementia versus MCI	≤17	65	65	0.76
	≤18	78	55	
	≤19	87	35	
	≤20	90	30	

Abbreviations: AUC, area under curve; MCI, mild cognitive impairment.

## 4 DISCUSSION

In a real‐world setting, we have shown that Free‐Cog has similar discriminatory power compared to other routinely used tests. It also proved to be sensitive and specific. The novel dual cognitive and executive function approach means the Free‐Cog complements existing assessment scales which measure these two related aspects of cognitive function. In the assessment of people with suspected dementia, all aspects of cognition are important, and it is acknowledged that global assessments (such as the Clinical Global Impression of Change Scales) are powerful tools which reflect the reality of the interdependency of both cognition and executive function. The limitation is that any specific effect on cognition may be diluted by the dual approach of this scale.

In developing the Free‐Cog, we have demonstrated that it is appropriate and feasible to combine cognitive and executive functions within one assessment. It appeared to be acceptable to patients and clinicians. We also established that it is possible to develop a scale which makes ecological sense. It is less likely to be perceived as a ‘test’, and performance may be less impaired by anxiety. The telephone consultation version, the Tele Free‐Cog was also able to discriminate between patient groups.

Larner^12^ compared the Free‐Cog and the Mini‐ACE (MACE) in a smaller study (N = 141) of patients referred to a speciality cognitive disorders clinic. Both tests had high sensitivity and large effect sizes for the diagnosis of dementia, but Free‐Cog was more specific. For the diagnosis of MCI, Free‐Cog lacked sensitivity (0.58) but was specific (0.81) whereas the MACE was sensitive (0.91) but not specific (0.35). A weighted comparison suggested equivalence for dementia diagnosis but a net benefit for MACE regarding MCI diagnosis.

There are limitations inherent to all cognitive assessment scales. For example, there is an inevitable relationship between the length of an assessment and its validity. Many concise scales are described as ‘screening’ instruments for dementia. This is mistaken, as screening for dementia is not justified using current criteria. Rather, these tools can be used for case finding and measurement of cognitive impairment, which then merits further investigation. Furthermore, the mathematical operationalisation of cognitive assessment tended to strip clinicians of the importance of the qualitative aspects of the psychiatric interview of which the cognitive examination is part. Scales are inherently reductionist, tending to describe people only in terms of a score, without the nuances of the patient's reactions. There is also inappropriate emphasis on cut offs to detect and define disease. Not all scales are directly translatable between cultures, and often require some degree of adaptation.^13^ For example, feedback from participants in this study suggested that some women may be hesitant to answer the functional domain which enquires about the steps they took to get dressed.

Whilst the Free‐Cog's performance is comparable to other similar scales, some important differences should be noted. We provide minimal written instructions for administration, designed to make it easier for busy clinicians to use. In addition, the executive function domains have a wider range of possible responses and are more subjective than the cognitive domains. This requires the assessor to use their judgement, and therefore it may not be suitable inexperienced operatives.

A key strength of this validation study is the sample. We recruited a large cohort of those attending outpatient services for older adults with memory and mental health complaints from centres across the United Kingdom. Therefore, our findings can be generalised to patients in similar healthcare settings. The current study has some limitations which should be noted. We did not assess interrater reliability or test–re‐test reliability. This is work should be carried out before the Free‐Cog is fully implemented into clinical practice. We also used the diagnoses given by clinical team in this analysis. A future study would benefit from using standardised diagnostic assessments to define patient groups. Assessors could not be blinded to clinical diagnosis or to the results of the comparator assessments. Furthermore, we were unable to control for the order in which tests were given, as we did not record this information.

## 5 CONCLUSION

We have developed a novel assessment tool which measure both cognitive and executive function. This large real‐world study suggests that the Free‐Cog is able to detect and measure cognitive impairment and is comparable to gold‐standard tools. It can add to the armamentarium of assessments available to clinicians. The Free‐Cog will remain freely available in perpetuity and we would welcome input from anyone interested in trialling it, translating it or further validating it. As a matter of courtesy, the authors would be interested to hear about these experiences at Alistair.Burns@manchester.ac.uk and we have plans to develop the website at https://www.gmmh.nhs.uk/free-cog.

## CONFLICT OF INTEREST

None.

## AUTHOR CONTRIBUTIONS

Alistair Burns and Catherine Symonds developed the scale and with Judith R. Harrison were involved in data collection. Julie Morris led on the statistical analysis. All the authors contributed to writing and redrafting the manuscript.

## Supporting information

Supplementary Material

## Data Availability

The data will be available subject to the usual IP regulations from our host institutions and in line with the journals policy.
