# Supplementary material for: A novel hybrid scale for the assessment of cognitive and executive function: The Free‐Cog
Source: Int J Geriatr Psychiatry. 2020 Nov 16;36(4):566–72. doi: 10.1002/gps.5454 (PMC7984170; doi:10.1002/gps.5454)
Supplement: Supplementary file 1 — Supplementary Material [file GPS-36-566-s001.docx]

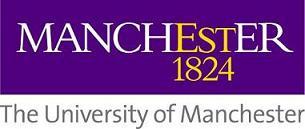
**Free Cog v1.0**
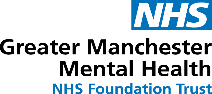


*IRAS Project ID : 227062*

*FREE COG Assessment v1.0 01.17*

| **Cognitive Function** | | |  | |
| --- | --- | --- | --- | --- |
| **Domain** | **Question/Instruction** | | **Score** | **Scoring guide** |
| **General Knowledge** | Can you tell me anything that’s in the news recently? | | **/1** | This is a general opening question to try to put people at ease, also accept recent events in sport or soap opera, if they state they don’t follow the news.  **Score 1 for any key fact of current knowledge but none for a general answer** |
| **Orientation (time)** | What day of the week is it today; what month, year? | | **/3** | Only allow accurate responses  **Score 1 point for each correct answer** |
| **Orientation (place)** | Where are we? | | **/3** | Only allow accurate responses  If in clinic/hospital score 1 for ward/ floor; 1 for hospital name; 1 for town  If in residence Score 1 for name/number of house; 1 for street name; 1 for town |
| **Memory (registration)** | Repeat 5 words  (watch, car, scarf, pen, house) | | **0** | **No score, record responses, allow up to 3 attempts.** |
| **Calculation** | Take 6 away from 70 and keep subtracting until I say stop | | **/3** | 64, 58, 52, 46, 40, then stop  **Score 3 = 5 or 4 correct; Score 2 = 3 or 2 correct**  **Score 1 = 1 correct; Score 0 = 0 correct** |
| **Attention** | Spell “plate” backwards | | **/2** | Check first they can spell “plate” then ask to spell it backwards  **Score 2 = 5 or 4 letters in correct order**  **Score 1 = 3 or 2 letters in correct order**  **Score 0 = 1 or 0 letters in the correct order** |
| **Memory (recall)** | Repeat 5 words  (watch, car, scarf, pen, house) | | **/5** | Record responses  **Score 1 point for each correct answer** |
| **Visuospatial** | In this circle draw a clock face with numbers and with hands set to ten past eleven | | **/3** | Draw a circle for the patient  **Score 1 point for each correct part:**  **All numbers present = 1 point**  **All numbers placed correctly = 1 point**  **Hands placed correctly = 1 point** |
| **Language** | Name ear and fingernail | | **/2** | Point to ear then fingernail  **Score 1 point for each correct answer** |
| **Fluency Task** | Name as many different animals as you can in 1 minute | | **/1** | Time one minute and record the responses  **Do not count different breeds of the same animal (eg corgi, spaniel, Cockapoo, Alsatian etc). Score 1 = if 10 or more correct responses**  **Score 0 = if <10 responses** |
| **Repeat a Sentence** | Repeat this sentence  **“**Don’t beat about the bush” | | **/1** | **Score 1 for repeated fully correctly** |
| **Write a Sentence** | Write a sentence | | **/1** | **Sentence needs to be understandable – ignore minor grammatical and spelling errors; Score 1 if fully correct** |
| **Executive Function** | | |  | |
| **Domain** | **Question** | | **Score** | **Scoring guide**  **These questions are to test the person’s ability to plan and describe sequences. Scoring is based on clinical judgement, non-leading clarifying questions may be asked if answers are tangential/ circumstantial** |
| **Social** | You have bought a birthday card and want to send it by post – tell me how you would do it? | | **/1** | **Score 1 = complete enough for the card to arrive**  **Score 0 = incomplete answer (i.e. card would not arrive)** |
| **Travel** | If you were going to take a bus (or train) what would you need? | | **/1** | **Score 1 = if answer indicates need for a ticket or bus pass**  **Score 0 = if they fail to mention ticket or bus pass** |
| **Home** | Could you tell me how you would make a cup of tea or coffee for yourself? | | **/1** | **Score 1 = if answer leads to a drinkable cup of tea/coffee**  **Score 0 = if answer does not lead to a drinkable cup of tea/coffee** |
| **Emergency** | If you discovered a fire at home, what would you do? | | **/1** | **Score 1 = if answer indicates the person would be appropriate and safe in their response; Score 0 = if they do not** |
| **Care** | Could you tell me the steps you took in order to get dressed as you are today? | | **/1** | **Score 1 = plausible story, consistent with the clothes they are wearing**  **Score 0 = incomplete and seems inconsistent with the clothing they are wearing** |
|  |  | **Total** | **/30** |  |
